# The Complete Chloroplast Genome of *Synotis solidaginea* and Phylogenetic Analysis

**DOI:** 10.1002/ece3.72806

**Published:** 2025-12-23

**Authors:** Yongming Fan, Le Chen, Yan Wu, Xiaohua Wang, Weili Tian, Meng Ge, Chu Li, Yaxin Xie

**Affiliations:** ^1^ School of Human Settlements North China University of Water Resources and Electric Power Zhengzhou China; ^2^ College of Landscape Architecture Beijing Forestry University Beijing China; ^3^ School of Electronic Engineering North China University of Water Resources and Electric Power Zhengzhou China; ^4^ Wellington Institute Zhengzhou University Zhengzhou China

**Keywords:** chloroplast genome, comparative genomics, phylogenetic analysis, SSR, *Synotis solidaginea*

## Abstract

*Synotis* is an important genus within the Asteraceae family. The genus comprises numerous species, many of which exhibit highly similar morphology, making identification using traditional taxonomic methods challenging. Therefore, developing a scientifically sound and effective identification method for *Synotis* plants is of significant importance. In this study, the complete chloroplast (cp) genome of *Synotis solidaginea* was sequenced using the Illumina HiSeq 4000 platform, analyzed, and compared with its closely related species. The results revealed that the complete chloroplast genome of *S. solidaginea* is 150,899 bp in length with a GC content of 37.47%. It exhibits the typical quadripartite structure, consisting of a large single‐copy (LSC) region (83,338 bp), a small single‐copy (SSC) region (17,885 bp), and two inverted repeat (IR) regions (each 24,838 bp). The genome encodes a total of 127 genes, including 83 protein‐coding genes, 36 tRNA genes, and 8 rRNA genes. Thirty‐two simple sequence repeats (SSRs) were identified. Four highly variable regions (*trnG‐UCC*, *ndhC‐trnV‐UAC_2*, *trnY‐GUA‐trnE‐UUC*, and *trnH‐GUG‐psbA*) were identified as potential DNA barcodes for species identification. Phylogenetic analysis revealed that *S. solidaginea* is closely related to its congeneric species *Synotis cavaleriei*, *Synotis erythropappa*, *Synotis duclouxii*, and *Synotis nagensium*. This study provides a reference for the phylogenetic analysis of *Synotis* using chloroplast genomics, and offers data support for the hybrid breeding of *S. solidaginea* to cultivate desirable ornamental traits or stress resistance traits. Furthermore, chloroplast genome research can also assist researchers in gaining a deeper understanding of the species origin of *S. solidaginea* and contribute to the study of species conservation and utilization.

## Graphical abstract

Species within the Asteraceae genus *Synotis* are difficult to accurately identify using traditional methods due to their highly similar morphology. In this study, the complete chloroplast genome of *Synotis solidaginea* was sequenced and comparatively analyzed. Four highly variable regions were identified as potential DNA barcodes, and phylogenetic analysis clarified its closely related species. This research provides genomic insights for species identification, phylogenetic studies, and breeding applications in the genus Synotis.

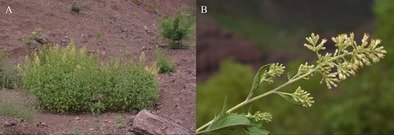

## 1 Introduction

*Synotis* is a genus of perennial herbs, shrubby herbs, or subshrubs belonging to the tribe Senecioneae of the Asteraceae family. The leaves of *Synotis* are typically simple, undivided, and range from broadly ovate‐cordate to narrowly oblong‐lanceolate. The genus comprises approximately 60 species. In China, all species except *Synotis atractylifolia* (distributed in the Helan Mountains of Ningxia) are confined to the Himalayan region. Chinese representatives of the genus are predominantly concentrated in the mountainous areas of the southwest (Chen et al. 2011). Furthermore, *Synotis* is also primarily distributed in Bhutan, India, Myanmar, Nepal, Thailand, and Vietnam (Liu et al. 2024).

The genus *Synotis* was previously classified under *Senecio* (groundsel genus), but was subsequently segregated as a distinct genus based on its anthers, which typically possess distinct tails at the base (Jeffrey and Chen 1984). Some species of *Synotis* bear a resemblance to those of the genus *Cissampelopsis*. However, *Synotis* comprises erect or slightly scandent herbs or subshrubs, lacking the characteristically thickened and cirrhose petioles found at the base of leaves in *Cissampelopsis*, making the two genera distinctly different. Their distribution ranges also differ significantly (Chen et al. 2011). Despite clear morphological distinctions between *Synotis* and its close relative *Cissampelopsis*, the phylogenetic relationships within *Synotis* and its affinities with related genera remain incompletely resolved (Liu et al. 2024). Traditional classification relies heavily on floral and vegetative traits. However, in topographically complex regions like the Himalayas, convergent evolution and phenotypic plasticity may obscure true evolutionary relationships (Brokaw and Hufford 2010; Zhang et al. 2022). Advances in molecular biology techniques have facilitated the use of markers such as the External Transcribed Spacer (ETS) (Li, Lazkov, et al. 2018), the Internal Transcribed Spacer (ITS) (Tong et al. 2017), and chloroplast DNA fragments in phylogenetic studies of *Synotis* (Tang 2014).

Chloroplasts are essential organelles within plant cells, primarily responsible for photosynthesis (Hippler et al. 2021). During this process, chloroplasts absorb light, carbon dioxide, and water to produce organic matter while releasing oxygen (Hippler et al. 2021). Research indicates that the biological functions of chloroplasts extend beyond photosynthesis; they are also involved in other critical biological processes. Chloroplasts act as a central regulatory hub in plant immunity through calcium signaling, reactive oxygen species bursts, hormone synthesis, and retrograde signaling, playing a vital role in plant metabolism, growth, and development (Rui et al. 2025). The chloroplast (cp) genome, as an independent genetic system, encodes approximately 100–130 genes. Among these, gene sequences and functions closely related to photosynthesis have remained relatively stable throughout evolution. This stability results in conserved regions within the cps of Asteraceae plants that are amenable to comparative analysis. Coupled with lower sequencing costs, cp genomes are increasingly becoming a valuable tool for studying plant phylogeny (Chen et al. 2023). Cp genomes exhibit relatively simple and stable structures with high conservation. They evolve at a slower rate compared to other plastid genomes and maintain greater structural stability, yet still display sufficient interspecific variation. Consequently, cp genomes are widely utilized in studies of plant population evolution, population genetics, and phylogenetic relationships, serving as an ideal tool for investigating evolutionary relationships among plant species (Fan et al. 2025; Odago et al. 2022; Wu et al. 2024; Zhang et al. 2017).

**FIGURE 1:**
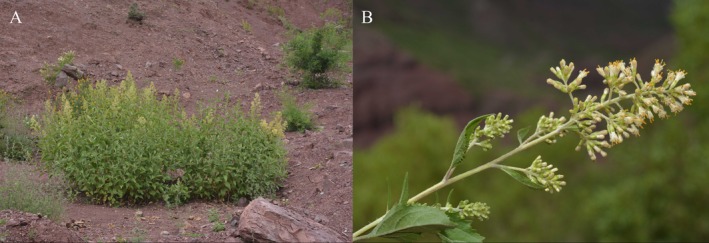
*Synotis solidaginea*. (A) plant habit; (B) leaf and inflorescence. Photo credit: Associate Research Professor Juntong Chen, Kunming Institute of Botany, Chinese Academy of Sciences.

*Synotis solidaginea* is a species within the genus *Synotis* (Asteraceae). Its leaves are ovate‐lanceolate, lanceolate, or elliptic‐oblong, with a cuneate or rounded base, and margins bearing dense sharply serrate or nearly double‐serrate teeth, sparsely covered with arachnoid pubescence (Figure 1). Of particular interest is that as a wild plant, *S. solidaginea* may harbor numerous valuable genes, particularly those conferring adaptability to harsh environments that have been lost in modern cultivated varieties. Investigating its chloroplast genome represents the foundational step toward comprehensively evaluating and utilizing its genetic resources. This effort will facilitate the discovery of unique alleles, thereby expanding the gene pool and providing candidate resources for future variety improvement. Currently, *S. solidaginea* has been studied extensively as a renowned Tibetan medicine, with numerous analyses focusing on its chemical constituents (Wei et al. 2024). However, research on its genetic mechanisms, evolutionary history, and developmental biology remains limited. The chloroplast genome, serving as a key tool for tracing maternal lineage, can be leveraged in comparative studies with congeneric or related species to reconstruct a well‐resolved phylogeny. This will clarify the evolutionary position of *S. solidaginea* within the Asteraceae family and even across angiosperms, thereby providing a theoretical basis for selecting hybridization parents. Furthermore, identifying highly variable regions in the chloroplast genome enables the development of abundant cytoplasmic molecular markers. These markers allow for rapid and accurate authentication of breeding materials or new varieties, helping to prevent misidentification and ensure genetic purity (Xu et al. 2017). Literature searches conducted in Web of Science and CNKI (China National Knowledge Infrastructure) revealed only six publications concerning *S. solidaginea* (as of July 9, 2025). This highlights a severe lack of breadth and depth in research on this species. Additionally, the cp genome sequence of *S. solidaginea* has not yet been published. Based on this, through sequencing the cp genome of *S. solidaginea*, it is possible to deeply analyze its genome structure, provide data support for understanding the plant's growth and development mechanisms, and reveal the genetic diversity and population structure of *S. solidaginea*. Through the analysis of the cp genome of *S. solidaginea*, important genetic resources can be provided for genomic breeding, aiding in variety improvement, especially in improving stress resistance, flower quality, and growth characteristics. In this study, the present study employed Illumina sequencing technology to determine the complete cp genome sequence of *S. solidaginea*. We characterized its genomic features and investigated the phylogenetic relationships between *S. solidaginea* and its closely related species.

## 2 Materials and Methods

### 2.1 Plant Material and DNA Extraction

Five leaves of *S. solidaginea* were collected in Déqìn County, Yunnan Province, China. No specific permissions were required for the collection of plant samples from this location. Voucher specimens are deposited at the Aquatic Plant Application and Germplasm Enhancement Laboratory of the Yellow River Basin, North China University of Water Resources and Electric Power, NCWU (Specimen number: NCWU‐20200627). Upon collection, the leaves were immediately stored at −20°C. Total genomic DNA was extracted from leaf tissue using the CretMag Multi Sample DNA Kit (Suzhou Cretaceous Biotechnology Co. Ltd., Suzhou, China) following a magnetic bead‐based protocol (Zhou et al. 2022). Upon quality assessment, the sample DNA showed an A260/A280 ratio of 1.78 (measured by NanoDrop), a concentration of 83.2 ng/μL (determined by Qubit), and clear main bands without significant degradation on agarose gel electrophoresis. In conclusion, the DNA quality met the requirements for subsequent library construction. Thereafter, a DNA library was constructed using the VAHTS Universal Plus DNA Library Prep Kit (Nanjing Vazyme Biotech Co. Ltd., Nanjing, China).

### 2.2 Sequencing, Complete Genomes Assembly, and Annotation

Sequencing was performed on the Illumina HiSeq 4000 platform (Shanghai OE Biotech. Co. Ltd., Shanghai, China) with 150‐bp paired‐end reads. Quality control of the raw sequencing data was conducted using FastQC v0.12.0 software (http://www.bioinformatics.babraham.ac.uk/projects/fastqc/, accessed: December 1, 2024) (Chen et al. 2018). The cp genome of *S. solidaginea* was assembled using GetOrganelle v1.7.7.1 (https://github.com/Kinggerm/GetOrganelle, accessed: March 18, 2025). The assembly was performed using the default settings of GetOrganelle v1.7.7.1 (Jin et al. 2020), with the cp genome of *Synotis cavaleriei* (NCBI accession: NC_069228.1) as the reference. Structural annotation of the chloroplast sequence was performed using CPGAVAS2 (http://www.herbalgenomics.org/cpgavas/) (Shi et al. 2019). The circular genome map was visualized using OGDRAW v1.2 (http://ogdraw.mpimp‐golm.mpg.de/, accessed: April 16, 2025) (Lohse et al. 2007).

### 2.3 Identification of Simple Sequence Repeats (SSRs)

SSR identification was performed using MISA (https://webblast.ipk‐gatersleben.de/misa/, accessed: March 18, 2025) (Beier et al. 2017). The minimum number of repetitions for SSR motif lengths 1, 2, 3, 4, 5, and 6 are 10, 6, 5, 5, 5, and 5, respectively.

### 2.4 Comparative Analysis of Cp Genomes

Comparative cp genome analysis was performed using mVISTA in Shuffle‐LAGAN mode (https://genome.lbl.gov/vista/mvista/submit.shtml, accessed: March 18, 2025) with *Senecio scandens* as the reference, comparing 11 closely related species (Frazer et al. 2004). Inverted repeat (IR) boundary visualization of *S. solidaginea* against 11 relatives was conducted using IRscope (https://irscope.shinyapps.io/irapp/, accessed: March 18, 2025) (Amiryousefi et al. 2018). Nucleotide diversity (Pi) was calculated using CPStools v2.5 (Huang et al. 2024). Based on previous phylogenetic analyses and taxonomic relevance, we selected 11 related species. The cp genomes of these species were downloaded from the NCBI database: *Anthriscus cerefolium* (accession number: NC_015113.1), *Crassocephalum crepidioides* (accession number: NC_057993.1), *Senecio drukensis* (accession number: PP525151.1), *Senecio graciliflorus* (accession number: PP525152.1), *Senecio raphanifolius* (accession number: PP525153.1), *Senecio scandens* (accession number: OQ475945.1), *Senecio thianschanicus* (accession number: PP525154.1), *S. cavaleriei* (accession number: NC_069228.1), *S. duclouxii* (accession number: NC_069229.1), *S. erythropappa* (accession number: NC_080520.1), *S. nagensium* (accession number: NC_069230.1).

### 2.5 Codon Usage Analysis

Codon usage bias analysis was performed using CodonW v1.4.2 (https://sourceforge.net/projects/codonw/files/codonw/Win32‐Executables‐1.4.2/, accessed: March 18, 2025), with calculation of relative synonymous codon usage (RSCU) values (Sharp and Li 1987). According to established standards, RSCU = 1.0 indicates no codon usage preference, RSCU > 1.00 signifies preferential codon usage (higher frequency).

### 2.6 Phylogenetic Analysis

Phylogenetic analysis was conducted using 56 complete cp genomes (accession numbers in Table S3), including the newly sequenced *S. solidaginea* genome from this study and 55 cp genomic sequences downloaded from the NCBI database. *A. cerefolium* and *A. sylvestris* were designated as outgroups. After extracting CDS using PhyloSuite v1.2.3, perform sequence alignment with MAFFT, Phylogenetic analyses were conducted using PhyloSuite v1.2.3 for comprehensive bioinformatics analysis and MAFFT v7.505 for initial multi‐sequence alignment, employing the “auto” strategy to select optimal algorithms for different sequence types. Sequence alignments were refined using trimAl v.1.2 to remove poorly aligned positions and divergent regions. For PCGs, batch refinement was performed using MACSE v2.0.3. Constructing a phylogenetic tree using the Maximum Likelihood method with IQ‐tree (http://www.iqtree.org/#download, Accessed March 18, 2025), bootstrap value of 5000 replicates (Katoh et al. 2019; Nguyen et al. 2015).

## 3 Results

### 3.1 Cp Genome Features of *S. Solidaginea*

**FIGURE 2:**
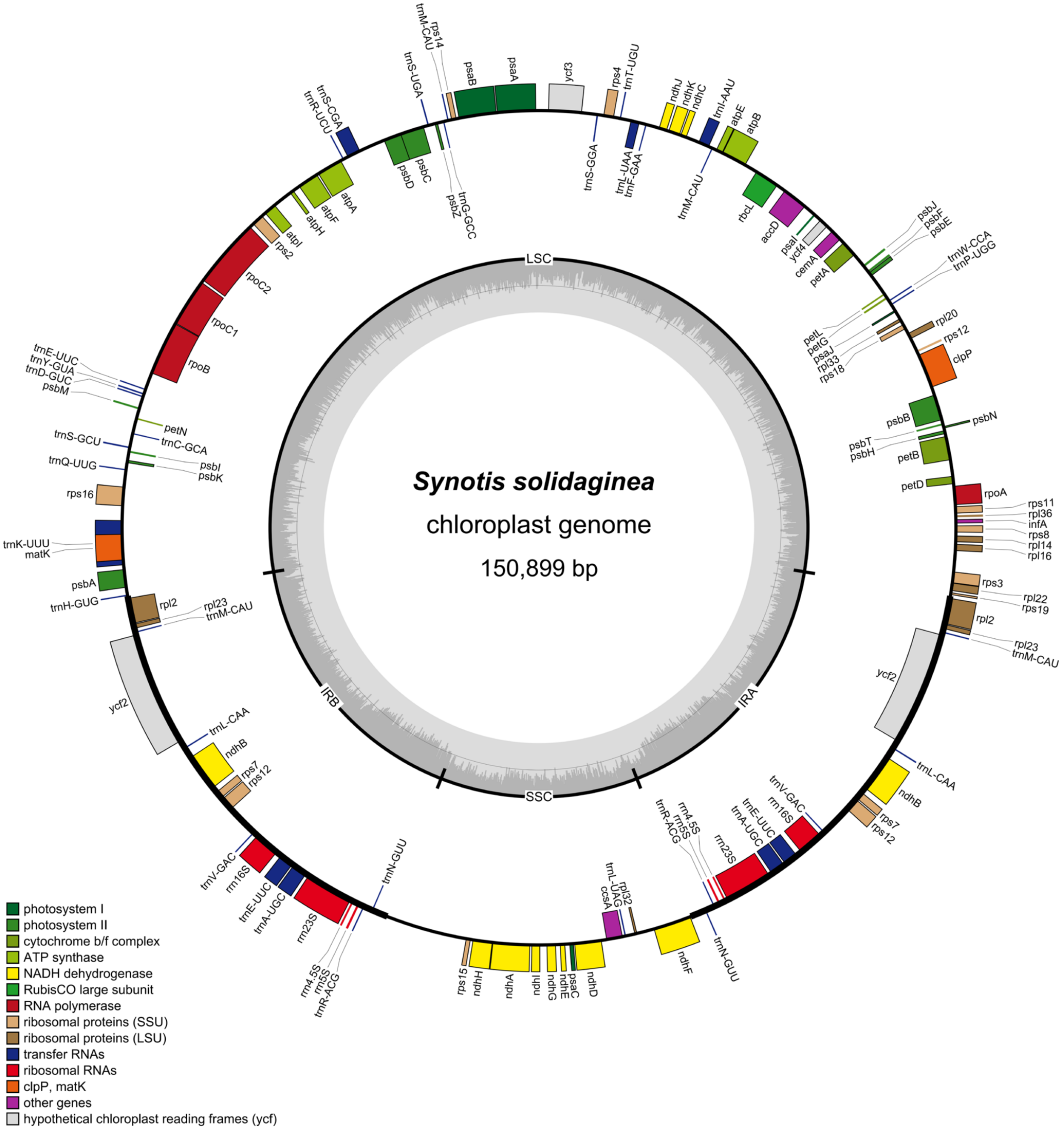
The circular complete cp genome map of *Synotis solidaginea*. Genes shown outside the outer circle are transcribed clockwise, whereas those shown inside are transcribed counterclockwise.

Sequencing generated 51,996,692 raw reads (7.80G raw bases). After quality filtering, 51,949,192 clean reads were obtained, totaling 7.75G clean bases, with 96.35% of bases above Q30 (proportion of bases with error rate < 1/1000). The cp genome of *S. solidaginea* is 150,899 bp in length. These cp genomes feature a typical quadripartite circular structure, including a large single‐copy (LSC) region (83,338 bp), a small single‐copy (SSC) region (17,885 bp) and two IR regions (24,838 bp each) (Figure 2). The overall GC content is 37.47%, with the IR regions exhibiting 43% GC content—higher than both the LSC region (35.61%) and SSC region (30.81%).

**FIGURE 4:**
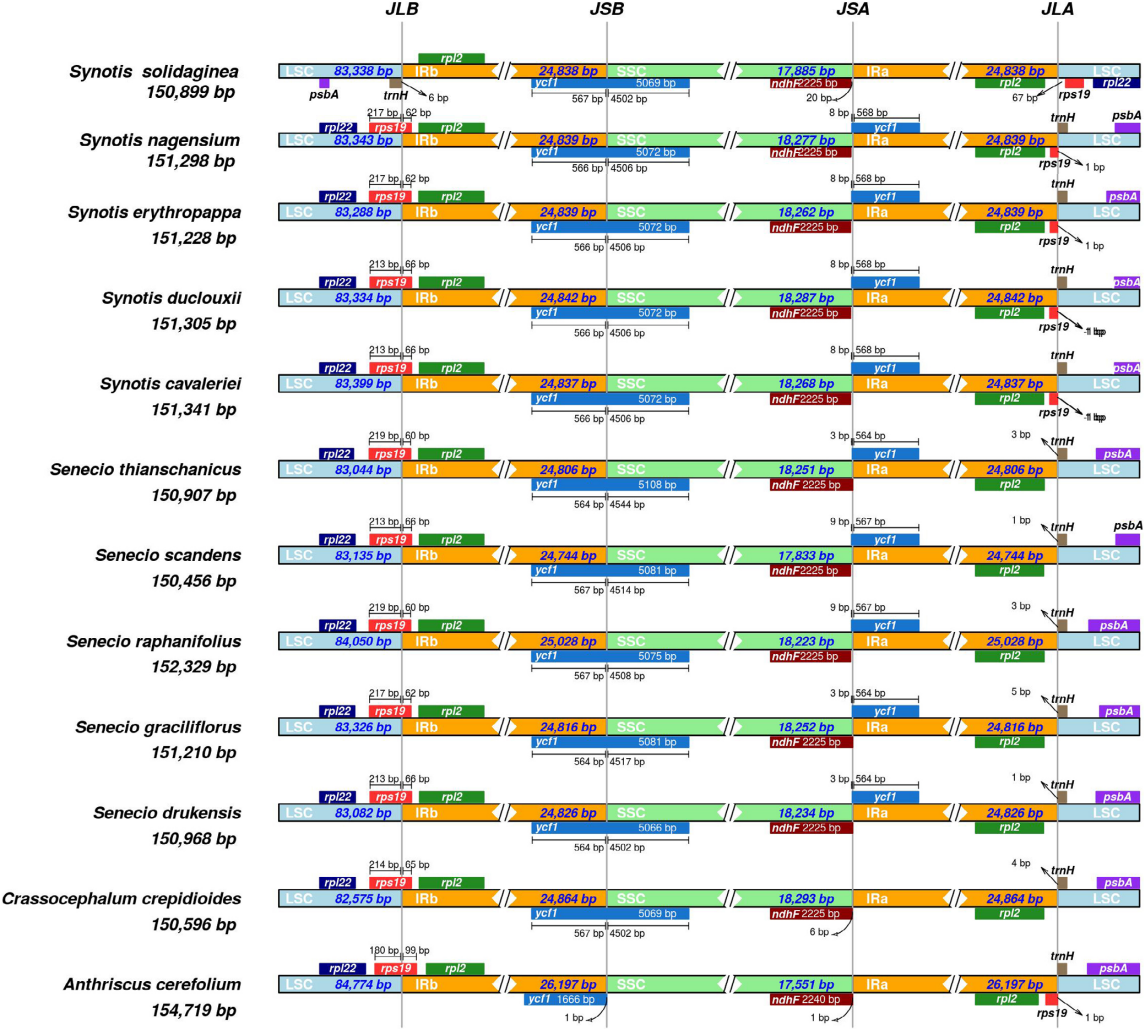
Comparison of the LSC, SSC, and IR regions among 11 selected cp genomes in the family Asteraceae. The gaps between the genes and the boundaries are indicated by the base lengths. Genes are denoted by colored boxes. JLB, Junction between LSC and IRb; JSB, Junction between IRb and SSC; JSA, Junction between SSC and IRa; JLA, Junction between IRa and LSC.

The cp genome of *S. solidaginea* contains 127 genes. Among these genes, 83 are protein‐coding genes, 36 are tRNA genes, and 8 are rRNA genes (Table S1. The chloroplast genome of *S. solidaginea* contains 127 genes). The chloroplast genes of *S. solidaginea* can be classified into four categories: photosynthesis, self‐replication, other genes, and unknown (Table 1). Among them, 17 genes are duplicated in the IR region, including 6 protein‐coding genes (*rpl2*, *prl23*, *ycf2*, *rps7*, *rps12*, and *ndhB*), 5 tRNA genes (*trnM‐CAU*, *trnL‐CAA*, *trnV‐GAC*, *trnR‐ACG*, and *trnN‐GUU*), and 6 rRNA genes (*rrn16s*, *trnE‐UUC*, *trnA‐UGC*, *rrn23S*, *rrn4.5S*, and *rrn5S*) (Figure 2). Due to the copy‐dependent repair via gene conversion between the two inverted repeat regions, the sequences of these duplicated genes are maintained with perfect identity. Nineteen genes (*rpl16*, *petD*, *petB*, *trnV‐UAC*, *trnL‐UAA, trnG‐UCC*, *atpF, rpoC1*, *rps16*, *trnK‐UUU*, *rpl2*, *ndhB*, *trnI‐GAU*, *trnA‐UGC*, *ndhA*, *trnA‐UGC*, *trnI‐GAU*, *ndhB*, and *rpl2*) contain only one intron, while two genes (*clpP* and *ycf3*) contain two introns (Table 2). The 5′ exon of the *rps12* gene is located in the LSC region, while the 3′ exon is located in the IR region, indicating that the *rps12* gene of *S. solidaginea* undergoes trans‐splicing (Figure 4). The cp genome sequence of *S. solidaginea* has been submitted to NCBI (accession number: PV916046).

**TABLE 1:** Gene composition in *Synotis solidaginea* cp genome.

Category of genes	Group of genes	Name of genes
Photosynthesis	Subunits of ATP synthase	*atpA, atpB, atpE, atpF* ^a^, *atpH, atpI*
	Subunits of photosystem II	*psbA, psbB, psbC, psbD, psbE, psbF, psbI, psbJ, psbK, psbM, psbN, psbT, psbZ, ycf3* ^b^
	Subunits of NADH dehydrogenase	*ndhA* ^a^, *ndhB* ^a^, ^c^, *ndhC, ndhD, ndhE, ndhF, ndhG, ndhH, ndhI, ndhJ, ndhK*
	Subunits of cytochrome b/f complex	*petA, petB* ^a^, *petD* ^a^, *petG, petL, petN*
	Subunits of photosystem I	*psaA, psaB, psaC, psaI, psaJ*
	Subunit of rubisco	*rbcL*
Self‐replication	Large subunit of ribosome	*rpl14, rpl16* ^a^, *rpl2* ^a^, ^c^, *rpl20, rpl22, rpl23* ^c^, *rpl32, rpl33, rpl36*
	DNA‐dependent RNA polymerase	*rpoA, rpoB, rpoC1* ^a^, *rpoC2*
	Small subunit of ribosome	*rps11, rps12* ^c^, *rps14, rps15, rps16* ^a^, *rps18, rps19, rps2, rps3, rps4, rps7* ^c^, *rps8*
Other genes	Subunit of acetyl‐CoA‐carboxylase	*accD*
	c‐type cytochrome synthesis gene	*ccsA*
	Envelope membrane protein	*cemA*
	Protease	*clpP* ^b^
	Translational initiation factor	*infA*
	Maturase	*matK*
Unknown	Conserved open reading frames	*ycf1, ycf15, ycf2* ^c^, *ycf4*

^a^ Gene containing a single intron.

^b^ Genes containing two introns.

^c^ Two gene copies in the IRs.

**TABLE 2:** The lengths of introns and exons for the splitting genes.

Gene	Strand	Start (bp)	End (bp)	ExonI (bp)	IntronI (bp)	ExonII (bp)	IntronII (bp)	ExonIII (bp)
*rpl16*	+	1571	3014	9	1036	399		
*petD*	−	6463	7663	9	718	474		
*petB*	−	7859	9268	6	762	642		
clpP	+	12,190	14,203	71	799	294	624	226
*trnV‐UAC*	+	31,591	32,238	38	573	37		
*trnL‐UAA*	−	35,818	36,344	37	440	50		
*ycf3*	+	39,197	41,140	124	697	230	740	153
*trnG‐UCC*	+	52,317	53,113	23	727	47		
*atpF*	−	55,148	56,400	145	698	410		
*rpoC1*	−	64,282	67,081	434	727	1639		
*rps16*	+	77,033	78,142	41	855	214		
*trnK‐UUU*	+	78,963	81,543	37	2509	35		
*rpl2*	−	83,454	84,967	397	686	431		
*ndhB*	−	93,369	95,572	777	671	756		
*trnI‐GAU*	+	101,087	101,935	42	772	35		
*trnA‐UGC*	+	102,000	102,893	38	821	35		
*ndhA*	+	114,442	116,608	553	1075	539		
*trnA‐UGC*	−	131,345	132,238	38	821	35		
*trnI‐GAU*	−	132,303	133,151	42	772	35		
*ndhB*	+	138,666	140,869	777	671	756		
*rpl2*	+	149,271	150,784	397	686	431		

*Note:* + Exon is transcribed counterclockwise in Figure 2; − exon is transcribed clockwise in Figure 2.

### 3.2 Analysis of SSRs

We identified 32 SSRs in the cp genome of *S. solidaginea* (Figure 3, Table S2. SSR in the *S. solidaginea* cp genome). These comprised 27 mononucleotide SSRs, 3 dinucleotide SSRs, 1 trinucleotide SSR, and 1 complex sequence repeat. Mononucleotide SSRs were the most abundant, accounting for 84.38% of the total. Dinucleotide SSRs constituted 9.38%, trinucleotide SSRs represented 3.12%, and complex sequence repeats also accounted for 3.12% (Figure 2).

**IGURE 3:**
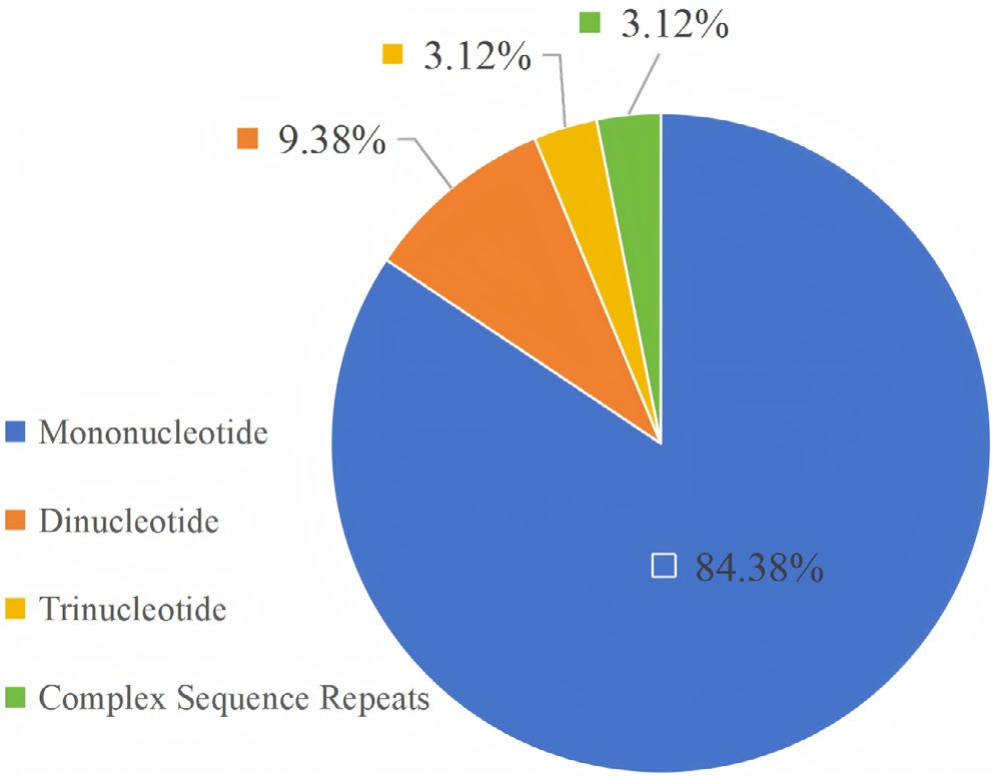
Frequencies of identified SSR types in *Synotis solidaginea*.

### 3.3 Comparative Analysis of Genome Structure in *S. solidaginea* and Its Related Species

The comparison analysis of the IR/SSC and IR/LSC boundaries can accurately pinpoint events of expansion and contraction of the inverted repeat sequences, revealing the dynamic evolutionary history of the chloroplast genome. To further elucidate the structural evolutionary history of *Synotis* cp genomes, we compared the IR/SSC and IR/LSC boundaries across 11 representative species of Asteraceae and one species of Apiaceae. These included *C. crepidioides* , *Senecio drukensis*, *Senecio graciliflorus*, *Senecio raphanifolius*, *Senecio scandens*, *Senecio thianschanicus*, *S. cavaleriei* , *S. duclouxii*, *S. erythropappa*, *S. nagensium*, and *A. cerefolium* (Figure 4). Genes involved in the IR/SSC and IR/LSC boundary regions included *ycf1*, *ndhF*, *rps19*, *rpl2*, *trnH*, *psbA*, and *rpl22*. Among *Synotis* and its close relatives, the IR region length ranged from 24,744 to 25,028 bp, exhibiting a certain degree of expansion.

In *Synotis nagensium*, *Synotis erythropappa*, *Synotis duclouxii*, *Synotis cavaleriei*, *Senecio thianschanicus*, *Senecio scandens*, *Senecio raphanifolius*, *Senecio graciliflorus*, and *Senecio drukensis*, the *ycf1* gene spanned the SSC/IRa boundary and was predominantly located within the IRa region. Simultaneously, the *ycf1* gene also spanned the IRb/SSC boundary and was predominantly located within the SSC region. In *S. solidaginea* and *Crassocephalum crepidioides* , the *ycf1* gene similarly spanned the IRb/SSC boundary and was predominantly located within the SSC region. In *A. cerefolium* , the *ycf1* gene spanned the IRb/SSC boundary but was predominantly located within the IRb region, this may be related to the length of the *ycf1* gene in *A. cerefolium* (Figure 4).

The *ndhF* gene was located at the SSC/IRa boundary in all 12 species, predominantly within the SSC region. With the exception of *S. solidaginea*, a functional copy of the *rps19* gene (conserved length of 279 bp) was present at the LSC/IRb junction in the other 11 species. The *rpl2* gene was completely located within the IR region and distant from both the LSC/IRb and IRa/LSC junctions in all 12 species (Figure 4).

The *trnH* gene was generally located within the LSC region. However, in *S. solidaginea*, *trnH* was positioned at the LSC/IRb junction, whereas in the other 11 species, it was located at the IRa/LSC junction. The *psbA* gene was generally located within the LSC region. In *S. solidaginea*, *psbA* was situated near the IRb region, while in the other 11 species, it was closer to the IRa region. The *rpl22* gene was generally located within the LSC region. In *S. solidaginea*, *rpl22* was situated near the IRa region, whereas in the other 11 species, it was closer to the IRb region (Figure 4).

**FIGURE 5:**
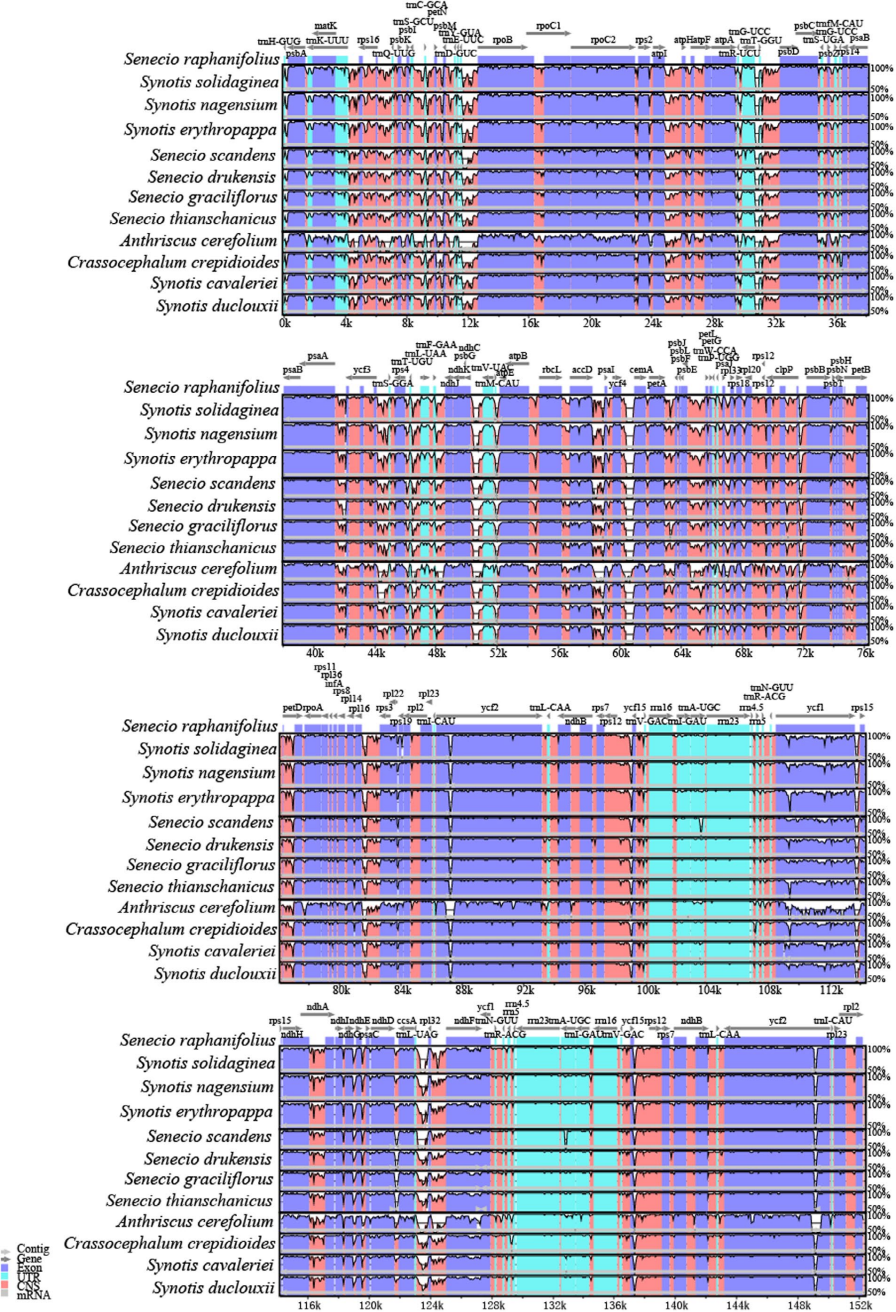
Sequence alignment of 11 Asteraceae cp. genomes using the mVISTA program with *Senecio raphanifolius* as a reference. *x*‐axis: The coordinates in the cp genome, *y*‐axis: Percent identity within 50%–100%. The transcriptional direction of genes indicated by gray arrows. Regions where the identity curve falls to the baseline (~50%) indicate sequences that are highly divergent or absent in the query genome. The gene names are indicated directly above the dark gray arrows.

Using *Senecio raphanifolius* as a reference, structural variations in the cp genomes of *S. solidaginea* and its related species were compared using the mVISTA software (Figure 5). The results revealed that the degree of variation was significantly higher in the LSC and SSC regions than in the two IR regions. Furthermore, noncoding regions exhibited considerably greater variability compared to coding regions.

### 3.4 Analysis of Codon Usage Bias in the Cp Genome of *S. Solidaginea*

The RSCU values were calculated based on the protein‐coding sequences within the cp genome of *S. solidaginea* (Table 3). The cp genome of *S. solidaginea* comprises 64 codons. Among these, 61 codons encode 20 amino acids, and 3 are stop codons. Within the *S. solidaginea* cp genome, leucine (Leu), arginine (Arg), and serine (Ser) were the most abundant amino acids, while methionine (Met) was the least abundant. Thirty‐three codons had RSCU values < 1 (RSCU < 1), indicating that their usage frequency was lower than the theoretical average under the assumption of no codon usage bias. Twenty‐eight codons had RSCU values > 1 (RSCU > 1), suggesting that their usage frequency was higher than the theoretical average. Methionine (Met) and tryptophan (Trp) had RSCU values equal to 1, showing no codon usage bia.

**TABLE 3:** Codon usage and codon‐anticodon recognition patterns of *Synotis solidaginea*.

Amino acid	Symbol	Codon	RSCU	Numbers
A	Ala	GCU	1.15	423
A	Ala	GCC	0.98	363
A	Ala	GCA	1.20	441
A	Ala	GCG	0.67	249
C	Cys	UGU	1.14	637
C	Cys	UGC	0.86	482
D	Asp	GAC	0.60	420
D	Asp	GAU	1.40	978
E	Glu	GAA	1.34	1336
E	Glu	GAG	0.66	658
F	Phe	UUC	0.81	1403
F	Phe	UUU	1.19	2045
G	Gly	GGA	1.42	786
G	Gly	GGC	0.64	357
G	Gly	GGG	0.99	549
G	Gly	GGU	0.95	529
H	His	CAC	0.69	462
H	His	CAU	1.31	874
I	Ile	AUA	1.00	1403
I	Ile	AUC	0.74	1040
I	Ile	AUU	1.25	1748
K	Lys	AAA	1.33	2088
K	Lys	AAG	0.67	1058
L	Leu	CUA	0.88	755
L	Leu	CUC	0.76	647
L	Leu	CUG	0.56	482
L	Leu	CUU	1.26	1079
L	Leu	UUA	1.35	1156
L	Leu	UUG	1.18	1011
M	Met	AUG	1.00	882
N	Asn	AAC	0.60	740
N	Asn	AAU	1.40	1735
P	Pro	CCA	1.27	714
P	Pro	CCC	0.98	548
P	Pro	CCG	0.70	394
P	Pro	CCU	1.05	586
Q	Gln	CAA	1.36	1083
Q	Gln	CAG	0.64	504
R	Arg	AGA	1.95	1038
R	Arg	AGG	1.14	605
R	Arg	CGA	0.99	530
R	Arg	CGC	0.50	266
R	Arg	CGG	0.77	411
R	Arg	CGU	0.65	348
S	Ser	AGC	0.68	489
S	Ser	AGU	0.90	654
S	Ser	UCA	1.16	840
S	Ser	UCC	1.10	795
S	Ser	UCG	0.73	525
S	Ser	UCU	1.43	1038
T	Thr	ACA	1.17	631
T	Thr	ACC	0.95	515
T	Thr	ACG	0.66	358
T	Thr	ACU	1.21	656
V	Val	GUA	1.19	654
V	Val	GUC	0.75	410
V	Val	GUG	0.67	370
V	Val	GUU	1.39	767
W	Trp	UGG	1.00	706
Y	Tyr	UAC	0.63	690
Y	Tyr	UAU	1.37	1494
*	Ter	UAA	1.20	1142
*	Ter	UAG	0.86	823
*	Ter	UGA	0.94	899

### 3.5 Nucleotide Diversity Analysis

The cp genome of *S. solidaginea* contains a substantial number of nucleotides. To gain deeper insights into the nucleotide diversity within its cp genome, we conducted sliding window analyses across these genomes and identified regions of high variability. We calculated nucleotide diversity (Pi) values for 104 coding genes and 94 intergenic spacer (IGS) regions across 11 species.

**FIGURE 6:**
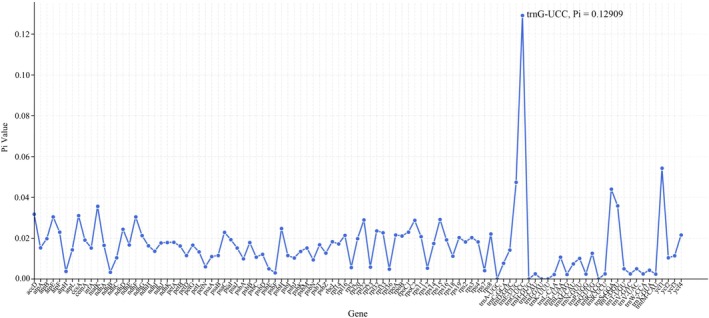
Sliding window analysis of cp genomes from 11 species. Nucleotide diversity (Pi) of intergenic regions in coding regions.

Within the coding regions, the nucleotide diversity (Pi) values ranged from 0.00000 to 0.12909 (trnG‐UCC), with a mean value of 0.01633. We identified one hypervariable regions (Pi > 0.12): trnG‐UCC (0.12909) (Figure 6). These discovered hypervariable loci hold potential as DNA barcodes for investigating the phylogenetic relationships between *S. solidaginea* and its closely related species or genera.

### 3.6 Phylogenetic Tree Construction and Analysis of Species Clusters

**FIGURE 7:**
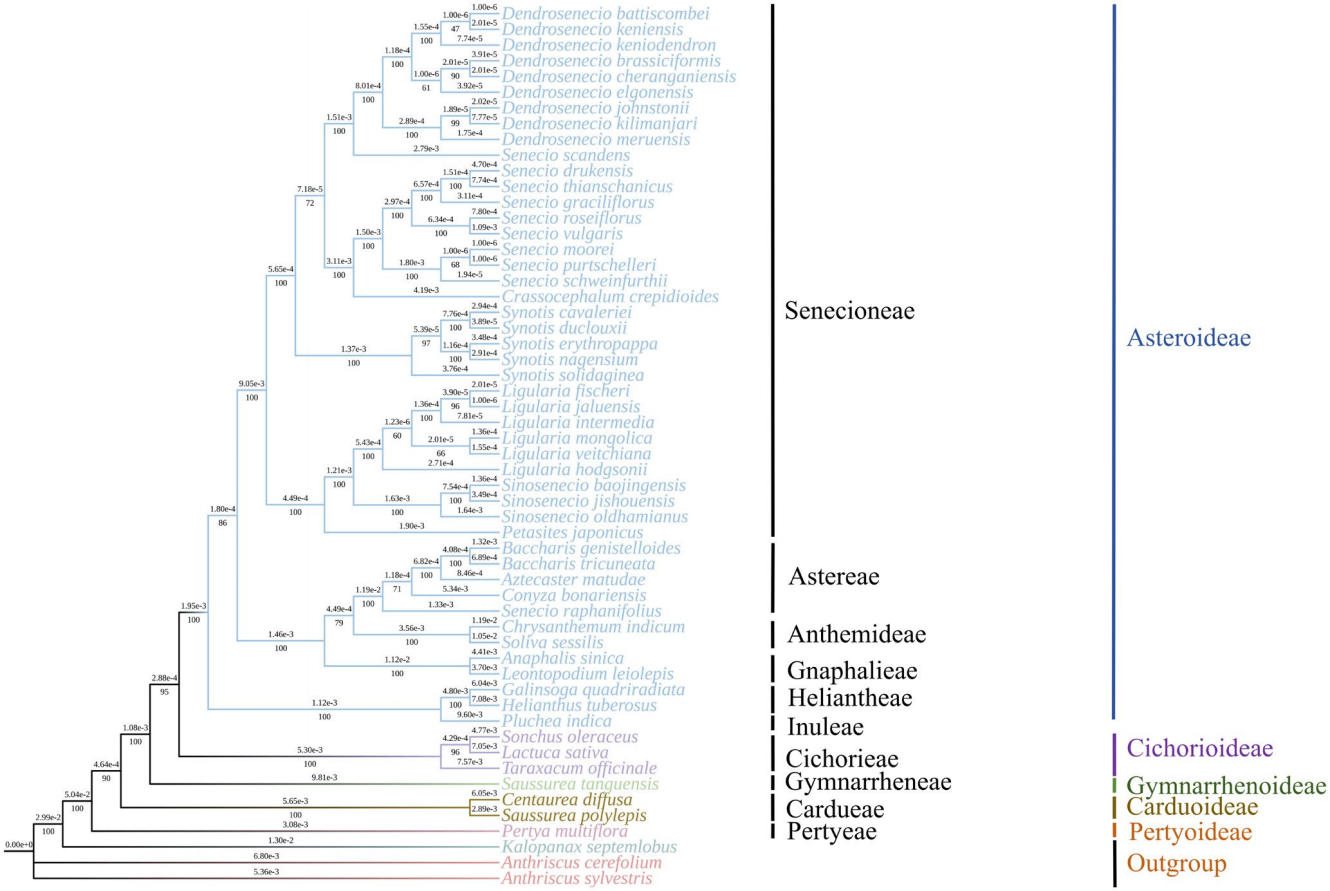
ML phylogenetic tree of 56 complete cp genomes. Bootstrap values and branch lengths are shown near each node. Different colors represent the differences in the clustering results.

Based on complete cp genome sequences, we constructed a phylogenetic tree for 54 Asteraceae species using the Maximum Likelihood (ML) method, with two Apiaceae species ( *A. sylvestris* and *A. cerefolium* ) as the outgroup (Figure 7). The bootstrap support values for the inferred phylogenetic relationships in the ML tree ranged from 60 to 100, indicating strong support for nearly all clades (Figure 7). In the species selected from the Asteraceae family, these species form a monophyletic group and are divided into five monophyletic clades, corresponding to the five subfamilies of Asteraceae. *Dendrosenecio battiscombei*, *D. keniensis*, *D. Keniodendron*, *D. brassiciformis*, *D. Cheranganiensis*, *D. elqonensis*, *D. johnstonii* , *D. kilimanjari*, *D. meruensis*, Senecio scandens, *Senecio drukensis*, *Senecio thianschanicus*, *Senecio qraciliflorus*, *Senecio roseiflorus*, *Senecio vulgaris* , *Senecio moorei*, *Senecio purtschelleri*, *Senecio schweinfurthii*, *Csrassocephalum crepidioides*, *S. cavaleriei* , *S. duclouxii*, *S. erythropappa*, *S. nagensium*, *S. solidaqinea*, *Liqularia fischeri*, *L. jaluensis*, *L. intermedia* , *L. mongolica* , *L. veitchiana*, *L. hodgsonii*, *Sinosenecio baojingensis*, *Sinosenecio jishouensis*, *Sinosenecio oldhamianus*, *Petasites japonicus* , *Baccharis qenistelloides*, *B. tricuneata*, *Aztecaster matudae*, *Conyza bonariensis* , *Senecio raphanifolius*, *Chrysanthemum indicum* , *Soliva sessilis* , *Anaphalis sinica*, *Leontopodium leiolepis*, *Galinsoga quadriradiata* , *Helianthus tuberosus* , *Pluchea indica* belong to the subfamily Asteroideae. *Sonchus oleraceus* , *Lactuca sativa* , and *Taraxacum officinale* belong to Cichorioideae. *Saussurea tanguensis* belongs to Gymnarrhenoideae. *Centaurea diffusa* and *Saussurea polylepis* belong to Carduoideae. *Pertya multiflora* belongs to Pertyoideae. The subfamily Asteroideae further includes the six tribes: Senecioneae, Astereae, Gnaphalieae, Anthemideae, Helenieae, and Inuleae. Within the genus *Synotis*, *S. solidaginea* clustered with its congeners *Synotis cavaleriei*, *Synotis duclouxii*, *Synotis erythropappa*, and *Synotis nagensium*, forming three distinct branches: (1) *Synotis cavaleriei* and *Synotis duclouxii* formed a clade with 100% support, (2) *Synotis erythropappa* and *Synotis nagensium* formed a clade also with 100% support, and (3) *S. solidaginea*formed a separate branch with 97% support (Figure 7). Furthermore, the phylogenetic tree revealed that *Crassocephalum* is closely related to Senecio, and Aztecaster is closely related to Baccharis and Conyza. *S. scandens* appears to be monophyletic with the genus *Dendrosenecio* rather than with *Senecio*, while *Senecio raphanifolius* occupies a highly divergent position on the phylogenetic tree, forming a clade with distantly related species (Figure 6).

## 4 Discussion

The cp genomes of land plants typically exhibit a highly conserved structure, often characterized by a typical quadripartite organization consisting of LSC and SSC regions, separated by two IR regions (Daniell et al. 2016). This study reports the first complete cp genome of *S. solidaginea*. We sequenced, assembled, and annotated the cp genome of *S. solidaginea* (Table 1 and Figure 2). Consistent with other angiosperms, the cp genome of *S. solidaginea* displays the typical quadripartite structure, comprising a large single‐copy region (LSC, 83,338 bp) and a small single‐copy region (SSC, 17,885 bp), separated by two inverted repeat regions (IRs, each 24,838 bp) (Figure 2). The complete cp genome length is 150,899 bp, similar to other Asteraceae species such as *Senecio drukensis* (150,968 bp; GenBank accession: PP525151.1, accessed July 5, 2025), *Senecio thianschanicus* (150,907 bp; PP525154.1, accessed July 5, 2025), and *Senecio vulgaris* (150,806 bp; NC_046693.1, accessed July 5, 2025). Analysis of sequenced cp genomes reveals variation in gene content, ranging from 63 to 209 genes, although most cp genomes contain between 110 and 130 genes (Jansen et al. 2005). The *S. solidaginea* cp genome contains 127 genes, including 83 protein‐coding genes, 36 transfer RNA (tRNA) genes, and 8 ribosomal RNA (rRNA) genes. The overall GC content is 37.47%, consistent with the gene count and GC content observed in most plant cp genomes (Liu et al. 2024; Gichira et al. 2019; Mahai et al. 2024). The GC content of the IR regions (43%) is higher than that of the LSC (35.61%) and SSC (30.81%) regions, similar to other Synotis species (Liu et al. 2024). Within the *S. solidaginea* cp genome, the *clpP* and *ycf3* genes each contain two introns, a feature also observed in the Asteraceae species *Artemisia maritima* and *Artemisia absinthium* (Shahzadi et al. 2020). The 5′‐end exon of the *rps12* gene is located in the LSC region, while its 3′‐end exon resides in the IR region. This genomic arrangement is consistent with findings in *S. cavaleriei* , *S. duclouxii*, *S. nagensium*, and *S. erythropappa* (Liu et al. 2024). In the cp genome of *S. solidaginea*, we identified genes related to photosynthesis, such as those in the Subunits of Photosystem II and Subunits of Photosystem I groups (Table 1). These findings provide important references for future research on the photosynthetic mechanisms in *Synotis*, while offering theoretical support for the genetic engineering of *Synotis* plants with higher photosynthetic efficiency and faster growth. This will help shorten the production cycle of *Synotis* in horticultural production.

We identified 32 SSRs within the *S. solidaginea* cp genome (Figure 3, Table S2). Among these, 27 were mononucleotide repeats, 3 were dinucleotide repeats, 1 was a trinucleotide repeat, and 1 was a complex sequence repeat. Mononucleotide SSRs were the most abundant, accounting for 84.38% of the total (Figure 3). These findings are consistent with those reported for the *Synotis* species *Synotis cavaleriei*, *Synotis duclouxii*, *Synotis nagensium*, and *Synotis erythropappa* (Liu et al. 2024). SSRs provide a powerful means of assessing genetic diversity within and among plant populations. It enables the detection of subtle genetic variations that are often undetectable in conserved chloroplast gene sequences, thereby offering critical discriminatory power for closely related taxa. Furthermore, SSR markers deliver unique information that complements and extends beyond the capabilities of other analytical methods, such as phylogenetic tree reconstruction and sequence alignment, making them an indispensable tool in population genetics and phylogenetic studies (Zhang et al. 2021; Li et al. 2020). In the breeding process of *Synotis* species, SSRs can be used for genotypic selection of target traits at the seedling stage, significantly shortening the breeding cycle and improving selection efficiency. Additionally, SSRs can also be used for purity identification of F_1_ hybrid seeds (Misiukevičius et al. 2023). This analysis of the SSR characteristics in the *S. solidaginea* cp genome provides a valuable reference for future research on molecular markers and genetic breeding in *Synotis* plants.

Comparative analysis of genome structure provides valuable scientific insights for in‐depth studies on the phylogeny and evolutionary relationships of Asteraceae species (Liu et al. 2024; Shahzadi et al. 2020). By comparing the IR/SSC and IR/LSC boundaries of *Synotis* with 11 representative Asteraceae species and one Apiaceae species, we observed a degree of expansion in the IR regions, with lengths ranging from 24,744 bp to 25,028 bp (Figure 4). This expansion phenomenon in the IR regions is similar to that reported in the Asteraceae genera *Saussurea* (Zhang et al. 2019), *Aster* (Tyagi et al. 2020), *Xanthium* (Raman et al. 2020), as well as in the *Synotis* species *Synotis cavaleriei*, *Synotis duclouxii*, *Synotis nagensium*, and *Synotis erythropappa* (Liu et al. 2024). In the present study, the *ycf1* gene in *S. solidaginea* spans the IRb/SSC boundary, with its major portion located within the SSC region, consistent with other *Synotis* plants. However, a distinguishing feature was observed: the *ycf1* gene in *S. solidaginea* does not span the SSC/IRa boundary, differing from other *Synotis* species (Liu et al. 2024). The *rpl2* gene is entirely located within the IR region and distant from both the LSC/IRb and IRa/LSC junctions, a characteristic consistent with other Asteraceae species (Gichira et al. 2019).

Codon usage bias in cp aids in the study of exogenous gene expression and cp evolution (Shen et al. 2024; Li et al. 2024; Sadhu et al. 2023). For *S. solidaginea*, we calculated the RSCU values, which reflect codon usage frequency across the cp genome. We identified 28 codons with RSCU values greater than 1 (RSCU > 1), indicating their usage frequency is higher than expected. Among these codons, the majority terminate with A or U (Table 3), a result consistent with findings in other angiosperms (Li, Li, et al. 2018; Li et al. 2019). We calculated the nucleotide variability values of 104 coding genes and 94 IGS regions across 11 species, and discovered 1 high‐variability regions (Pi > 0.12) in *S. solidaginea*, including *trnG‐UCC* (0.12909) (Figure 6). These high‐variability regions can serve as specific DNA barcodes for studying the phylogenetic relationships between *S. solidaginea* and its closely related species or genera (Daniell et al. 2021). In phylogenetic and DNA barcoding research, cp DNA is suitable for comparing distantly related species and analyzing degraded materials due to its uniparental inheritance, structurally conserved nature, and high copy number. However, its conservatism limits the resolution for closely related species. While nuclear DNA markers offer higher resolution at lower taxonomic levels, they are constrained by issues such as paralogy and heterozygosity (Li et al. 2021). This study identified highly variable regions (*trnG‐UCC*) in the chloroplast genome of Synotis solidaginea, confirming a gradient of evolutionary rates across different regions of the chloroplast. Therefore, combining chloroplast and nuclear DNA markers is crucial for studying the evolutionary history of complex species.

Based on complete cp genome sequences, with the Apiaceae plants *A. sylvestris* and *A. cerefolium* as outgroups (Figure 7), we constructed a phylogenetic tree of 54 Asteraceae species. The results show that *S. solidaginea* forms a sister group with its congeneric species *Synotis cavaleriei*, *Synotis duclouxii*, *Synotis erythropappa*, and *Synotis nagensium* (Figure 7). Previous research revealed that *Synotis cavaleriei* and *Synotis duclouxii* cluster together, and *Synotis nagensium* and *Synotis erythropappa* cluster together, which is consistent with the results of this study (Liu et al. 2024). *S. solidaginea* forms a separate branch and is a sister group to the four congeneric species, indicating their close genetic relationship, consistent with traditional taxonomy results (Chen et al. 2011). Furthermore, the separation of *S. solidaginea* from its congeneric species also proves that its complete cp genome can serve as a specific DNA barcode for studying the phylogenetic relationships between *S. solidaginea* and its closely related species or genera. The phylogenetic tree revealed that *Crassocephalum* is closely related to *Senecio*, consistent with previous research results (Zhang and Gong 2023). *Aztecaster* is closely related to *Baccharis*, consistent with the findings of Xie et al. (2021). *S. scandens* appears to be monophyletic with the genus *Dendrosenecio* rather than with *Senecio*, possibly due to nuclear‐cytoplasmic discordance. This suggests that hybridization and introgression may have occurred between *S. scandens* and an ancestor of *Dendrosenecio*, resulting in its chloroplast genome being derived from *Dendrosenecio*, while its nuclear genome remains primarily from *Senecio*. This would lead to conflict between phylogenies based on chloroplast data and those based on nuclear genes. The data for *Senecio raphanifolius* sourced from NCBI may exhibit this phenomenon due to significant differences in sequencing technologies. Furthermore, phylogenetic analysis provides scientific evidence for evaluating the genetic background of wild germplasm resources, aiding in the guidance of *Synotis* hybrid crossbreeding in horticultural breeding to avoid inbreeding depression (Duan et al. 2022). At the same time, wild plants have evolved strong resistance mechanisms to adapt to their environment. Many resistance‐related proteins are encoded by chloroplast genes or by nuclear genes and then function in the chloroplast (Tang et al. 2025). Phylogenetic analysis of wild species *S. solidaginea* offers important references for further exploring their valuable genes related to disease resistance and stress tolerance, making them an important resource for horticultural breeding.

## 5 Discussion

This study reports for the first time the complete cp genome of *S. solidaginea*, with a size of 150,899 bp. The cp genome of this species contains 127 genes, including 83 protein‐coding genes, 36 tRNA genes, and 8 rRNA genes. Phylogenetic analysis found that S. solidaginea forms a sister group with its congeneric species *S. cavaleriei* , *S. erythropappa*, *S. erythropappa*, and *S. nagensium*, indicating a close genetic relationship. The complete cp genome of *S. solidaginea* can serve as an important basis for identifying *Synotis* species.

## Author Contributions

**Yongming Fan:** conceptualization (lead), investigation (lead), methodology (equal), project administration (equal), resources (equal), validation (equal), writing – original draft (lead), writing – review and editing (lead). **Le Chen:** data curation (equal), formal analysis (equal), software (equal), validation (equal), visualization (equal), writing – original draft (equal), writing – review and editing (equal). **Yan Wu:** data curation (equal), methodology (equal), validation (equal), writing – original draft (equal), writing – review and editing (equal). **Xiaohua Wang:** conceptualization (equal), data curation (equal), formal analysis (equal), writing – original draft (equal), writing – review and editing (equal). **Weili Tian:** data curation (equal), writing – original draft (equal), writing – review and editing (equal). **Meng Ge:** data curation (equal), writing – original draft (equal), writing – review and editing (equal). **Chu Li:** data curation (equal), visualization (equal), writing – original draft (equal), writing – review and editing (equal). **Yaxin Xie:** data curation (equal), funding acquisition (lead), project administration (lead), writing – original draft (equal), writing – review and editing (equal).

## Funding

This research was supported by the Natural Science Foundation of Henan (Grant number 252300423627), the Graduate Education Reform Project of Henan Province (Grant number 2023SJGLX113Y), and the High‐level Talent Scientific Research Startup Project of North China University of Water Resources and Electric Power (Grant number 202110007).

## Conflicts of Interest

The authors declare no conflicts of interest.

## Supporting information


**Table S1:** The chloroplast genome of *Synotisa solidaginea* contains 127 genes.

**Table S2:** Simple sequence repeats (SSR) in the *Synotisa solidaginea* cp genome.

**Table S3:** Accession numbers of 56 complete chloroplast genomes used for phylogenetic analysis.

## Data Availability

The chloroplast genome sequence of *S. solidaginea* has been submitted to NCBI (accession number: PV916046).
